# Supplementary material for: MetaRibo-Seq measures translation in microbiomes
Source: Nat Commun. 2020 Jun 29;11:3268. doi: 10.1038/s41467-020-17081-z (PMC7324362; doi:10.1038/s41467-020-17081-z)
Supplement: Supplementary file 10 — Supplementary Data 7 [file 41467_2020_17081_MOESM10_ESM.zip › File2/Confidence_High_Taxonomy/386912_out.krona.html]

Javascript must be enabled to view this page.

members
magnitude
magnitudeUnassigned
count
unassigned
taxon
rank

386912\_out

3

2
superkingdom
3

phylum
3
1239

909932
3
class

3
order
1843488

909930
3
family

33024
genus
3

33025
2

SRS012273\_contig\_number\_32239SRS077849\_contig\_number\_18807
species

1262914

SRS020394\_contig\_number\_3396
species
1
